# Vitellogenin from the Silkworm, *Bombyx mori*: An Effective Anti-Bacterial Agent

**DOI:** 10.1371/journal.pone.0073005

**Published:** 2013-09-13

**Authors:** Nitin Kumar Singh, Britto Cathrin Pakkianathan, Manish Kumar, Tulika Prasad, Mani Kannan, Simone König, Muthukalingan Krishnan

**Affiliations:** 1 Department of Environmental Biotechnology, Bharathidasan University, Tiruchirappalli, India; 2 Advanced Instrumentation Research Facility, Jawaharlal Nehru University, New Delhi, India; 3 Integrated Functional Genomics, Interdisciplinary Center for Clinical Research, University of Münster, Münster, Germany; Instituto Butantan, Brazil

## Abstract

Silkworm, *Bombyx mori*, vitellogenin (Vg) was isolated from perivisceral fat body of day 3 of pupa. Both Vg subunits were co-purified as verified by mass spectrometry and immunoblot. Purified Vg responded to specific tests for major posttranslational modifications on native gels indicating its nature as lipo-glyco-phosphoprotein. The Vg fraction had strong antibacterial activity against Gram negative bacterium *Escherichia coli* and Gram positive bacterium *Bacillus subtilis*. Microscopic images showed binding of Vg to bacterial cells and their destruction. When infected silkworm larvae were treated with purified Vg they survived the full life cycle in contrast to untreated animals. This result showed that Vg has the ability to inhibit the proliferation of bacteria in the silkworm fluid system without disturbing the regular metabolism of the host.

## Introduction

The Mulberry silkworm, *Bombyx mori,* is not only of enormous commercial importance for silk production, but it also serves as research model organism for studying Lepidopteran insects that cause serious agricultural damage (for brief overview see [1]). The lipid transfer protein vitellogenin (Vg) is the precursor of egg yolk proteins in the silkworm, *B. mori,* [2] and constitutes one of the major proteins in silkworm. A review on the research regarding hemaglutinating and antibacterial activities of this molecule may be recommended for introductory reading [3]. It discusses functions of Vg not only as an energy reserve for the developing embryo but also in innate immune response. Vg has been studied in insects since the 1960ies [4]. The respective work in silkworm traces back to the Science publication by the Telfer group which postulated female-specificity of Vg production in moth and roach fat body (FB) [5]. This group also looked at silkworm [6] and thereby triggered Vg research in *B. mori*. The Vg gene sequence of *B. mori* was published in 1994 ([2], [7], Uniprot accession number Q27309, VIT_BOMMO, molecular weight (MW) 203055 Da). In a gel electrophoretic study of hemolymph proteins female Vg of *B. mori* was detected at ∼178 kDa in the early non-feeding (pupal) stage [8]. *B. mori* Vg was described as tetramer (MW 403116 Da [2], [7]) composed of two molecules each of heavy and light chains (amino acid residues (AA) 16–370, vitellin light chain Vg-L, MW 40229 Da; AA 371–1782, vitellin heavy chain Vg-H, MW 161329 Da) although the subunits seemed to be encoded by a single continuous mRNA (Note ambiguity about the terms Vg and vitellin: The precursor is often referred to as Vg, the yolk protein as vitellin [4]). In wild silkworm, *Antheraea pernyi,* and *Saturnia japonica*, Vg was purified as dimer at a MW of ∼210 and ∼200 kDa, respectively [9], [10]. Intracellular proteolytical processing of Vg precursor protein translated from the mRNA of *B. mori* to Vg-H and Vg-L chain was linked to subtilisin-like convertase [11] and placed at the end of the larval period, before uptake by developing oocyte [2], [12], [13]. In the fish *Oreochromis aureus*, the N-terminus of Vg was identified as the Vg receptor binding site [14] and this was also reported for honeybee Vg [15]. Vg-L has the large lipid transfer (LLT)-domain common to all lipoproteins of this superfamily [16].

Functions of Vg involve reduction of oxidative stress [17], [18] and regulation of hormonal dynamics [19], [20]. Recently, piscine Vg has been shown to be a multivalent pattern recognition molecule capable of identifying non-self components including lipopolysaccharides, peptidoglycans, lipoteichoic acid and glucan and to act as an opsonin that can enhance macrophage phagocytosis [21], [22]. Such functional information is still missing for silkworm. We purified and characterized *B. mori* Vg in particular regarding antimicrobial activity, which has been described in addition to hemagglutinating activity in protochordate amphioxus *Branchiostoma belcheri* [23], in the bony fish rosy barb *Puntius conchonius* and carb [22], [24], [25], salmon [26] as well as chicken [27]. Mosquito Vg was shown to modify the anti-Plasmodium response in *Anopheles gambiae* [28]. Hence, in the present study Vg was isolated from *B. mori* strain, Tamil Nadu×NB4D2, and investigated with respect to its properties.

## Materials and Methods

Unless otherwise noted, sample collection and experiments (gel electrophoresis, Western blotting, lipoprotein staining of native gel) were performed as described earlier [29]. The monoclonal antibody against Vg of *Pteromalus puparum* was a generous gift from Prof. Gong-Yin Ye; State Key Laboratory of Rice Biology, Institute of Insect Sciences, College of Agricultural and Biotechnology, Zhejiang University, China. Insect developmental stages (Note: e.g. “3rd day of the 5th instar larvae” is abbreviated as “day 3 V instar”) were synchronized at each molt by collecting new larvae.

### Ion exchange chromatography

DEAE-cellulose (2 g, Sigma) was activated as described [30]. The matrix was equilibrated with 0.1 M Tris citrate buffer (pH 7.3) and packed into a glass column (2 cm×22 cm; Borosil, India). Crude protein sample (3 mg) collected from day 3 of pupal perivisceral (PV)FB was loaded onto the column and was washed with equilibration buffer for 2 h to remove unbound proteins. Bound proteins were eluted using a linear gradient of NaCl solution increasing the concentration from 0 to 0.5 M at a flow rate of 1 ml/3 min. A total of 250 fractions were collected. Protein samples (15 μg) were subjected to 7% sodium dodecyl sulfate polyacrylamide gel electrophoresis (SDS-PAGE) and gels were silver stained [31]. The fractions containing only three bands at ∼200, 180 and 46 kDa were tested for Vg by immunoblot analysis, pooled and stored at −80°C for further analysis.

### Phosphoprotein staining

[32] Native gels were exposed to 10% sulfosalicylic acid (SSA, w/v) overnight. They were transferred to 10% SSA solution containing 0.5 M CaCl_2_ for 1 h followed by washing in water twice. Subsequently gels were immersed in 0.5 N NaOH at 60°C for 30 min followed by washing in ammonium molybdate solution twice. Gels were moved into a 1% aqueous ammonium molybdate solution (w/v) containing 1 N HNO_3_ for 30 min. Finally, gels were stained with 0.5% methyl green (w/v) diluted in 7% acetic acid solution (v/v) for 30 min. Destaining was carried out in 10% SSA at 60°C and gels were stored in 7% acetic acid solution until they were dried for storage.

### Glycoprotein staining

[33] Native gels were incubated in 12.5% trichloroacetic acid (w/v, 30 min) and then rinsed with double-distilled water. Subsequently they were immersed in 1% periodic acid (w/v), prepared in 3% acetic acid (v/v) for 50 min. They were washed with double-distilled water overnight to remove excess periodic acid. Gels were transferred to Schiff’s reagent and kept in the dark for 50 min. Gels were washed with freshly prepared 0.5% potassium metabisulfphite (w/v) thrice for 10 min each. Then gels were washed with double-distilled water to remove excess stain and stored in 7% acetic acid until they were dried for storage.

### Mass spectrometry (MS)

Matrix-assisted laser desorption/ionization time-of-flight (MALDI- TOF) MS was carried out using Ultraflex (Bruker Daltonics, Germany) in reflectron mode. Peptide masses were recorded and analyzed at 300 ppm mass accuracy with the online software tool MASCOT (Matrix Science, Inc.) for protein identification using NCBI and UniProt public databases.

### Analysis of Vg effect on bacterial growth

Five different concentrations (50, 75, 100 and 125 µg/ml) of purified Vg were used to test antibacterial activity against *Escherichia coli* (MTCC-739) and *Bacillus subtilis* (MTCC 441) purchased from the Institute of Microbial Technology, Chandigarh, India. Bovine serum albumin (BSA) at the same concentrations (50, 75, 100 and 125 µg/ml) was used as control. Aliquots were inoculated with 0.1 ml (5×10^7^ cells/ml as determined by hemocytometer) of overnight grown *E. coli* and *B. subtilis* cells in nutrient broth [34]. Inoculated cultures were supplemented with Vg or BSA, respectively, and incubated at 37°C for 24 h. Every four hours the turbidity of nutrient broth was measured at 420 nm. Experiments were carried out in triplicate.

### Antibacterial assay

Four different concentrations (1, 2, 3, 4 µg/µl) of purified Vg were used with ampicillin (40 µg in 10 µl solution) as reference. Sterile Muller-Hinton agar medium was poured into sterilized standard Petri dishes and allowed to solidify. Overnight grown *E. coli* or *B. subtilis* cells (0.1 ml) were swabbed onto the agar plates. Sterile paper discs (Hi-media, India) were placed on the bacterial lawns. Vg was applied onto the discs with the reference antibiotic at the centre disc. The plates were incubated at 37°C overnight. Zones of growth inhibition around the paper discs were measured with a graduated scale. Experiments were carried out in triplicate.

### FITC-labelling

Purified Vg and BSA (0.1 mg each) were labeled with FITC (Genei, India) following the manufacturer's instructions. The labelled protein was separated from free FITC by exclusion gel chromatography (Sephadex G-25). Fractions were collected and their absorbance at 280 nm was measured. Fractions with an optical density (OD) value >0.6 were taken for further experiments. The labelled protein aliquots were stored at −80°C.

### Binding of FITC labelled Vg to microbial cells

Aliquots of 30 µl of FITC-labelled Vg or BSA (0.2 mg/ml) were mixed with 30 µl *E. coli* or *B. subtilis* (5×10^7^ cells/ml) and incubated at 4°C overnight. Microbial suspension (5 µl) was applied to microscope slides and studied using fluorescent microscope Nikon Trinocular E200 (435 barrier filter, 365 excitation filter).

### Scanning electron microscopy (SEM)

Aliquots of 150 µl of *E. coli* and *B. subtilis* suspension containing 3×10^7^ cells/ml were mixed with 0.1 mg purified Vg protein or BSA in 0.1 M Tris citrate buffer (pH 7.3). For control, overnight cultured cells were mixed with 150 µl Tris citrate buffer. The mixtures were incubated at 25°C for 4 h and then fixed in 2.5% glutaraldehyde/100 mM PBS (pH 7.4). Fixed cells were washed with 100 mM PBS, post-fixed with 0.1% osmium tetraoxide and dehydrated with graded ethanol. The samples were dried with hexamethyldisilizane and coated with gold (15 mm). The coated samples were spread on microscope slides and investigated using Zeiss Ev040 scanning electron microscope.

### Therapeutic effects of Vg protein against bacterial diseases of silkworm

Larvae (30 insects of day 1 V instar) were infected with 1 µl (1×10^7^ cell forming units/ml) of bacterial suspension containing both *E. coli* and *B. subtilis* at equal amounts. Half (15 insects) of the infected silkworms were treated with 200 µg purified Vg protein in 50 µl milliQ water by microinjection into the first mid leg. Pressure was immediately applied at the injection site using a finger for 15 s to stop bleeding. Control larvae (15 insects) were pricked with a sterile entomological pin. The experiments were replicated five times. For each replicate the total number of larvae vital by the end of the experiment was recorded (Table S2). The larvae were maintained at room temperature with the normal rearing procedure. Hemolymph for two-dimensional (2D)-PAGE was collected 24 h post injection.

## Results and Discussion

### Purification and confirmation of Vg protein

Protein purification is the prerequisite to study its function. The best response for Vg by immunoblot analysis with Vg specific primary antibody was observed in perivisceral fat body (PVFB) and hemolymph proteomes starting at day 3 of V instar larvae (Figure S1). Signals were detected at ∼200/180 and ∼46 kDa supposedly for Vg precursor and its light and heavy chains up to pupa day 6 (data not shown). Unpublished radiolabelling studies by Vanishree [35] had already suggested Vg production in PVFB. Therefore, Vg protein isolation was carried out using day 3 of pupal PVFB tissue. Detailed investigations to clarify stage-dependent Vg biosynthesis are in progress.

Previous studies on *Apis mellifera*, *Manduca sexta, Spodoptera litura*, and recently on *Oscheius tipulae* used ion exchange chromatography to purify Vg; however, they have employed additional separation procedures such as density gradient ultracentrifugation and affinity chromatography to remove contaminating protein [15], [36]–[39]. Here, Vg protein was isolated from 3 mg of total protein from day 3 of pupal PVFB in a single-step procedure by optimizing buffers with respect to the pI 6.5 of Vg. Vg purification from FB was tedious, because the samples contained many lipoproteins, storage proteins and 30 kDa proteins. Purity control of the fractions of interest was performed with SDS-PAGE and silver staining (Figure S2). A number of fractions (35–115) exhibited Vg proteins. Three major bands were visible at ∼46 kDa supposedly for Vg-L and at ∼180 and 200 kDa for Vg-H and Vg precursor, respectively, in fractions 110–114. With elution of the precursor only two bands appeared in fraction 115. Western blot analysis with Vg specific antibody supported this hypothesis.

In native PAGE, the Vg proteins were located in the same MW regions (Figure S2B) indicating little influence of preparation steps (involving, e.g., SDS or mercaptoethanol) on protein folding and complex formation. Obviously, two or three Vg protein forms were co-purified by the anion-exchange procedure pointing to their co-presence in PVFB. Earlier reports [40], [41] found native Vg of honeybee with an additional weight of ∼10 kDa which was contributed to carbohydrate and lipid moieties. Fish Vg had been separated on native PAGE at ∼450 kDa [21], [22]. In many lepidopteran insects, Vg is synthesized as a single protein that is cleaved into heavy and light chains [11].

The presence of Vg was confirmed by MS peptide mapping. Database search matched peptides derived from the UniProt VIT_BOMMO entry for both bands. Signals covered the C-terminal region up to AA 285 for the 46 kDa band and the C-terminal range starting from AA 373 for the 180 kDa band. Vg has been described as high density phospho-lipo-glycoprotein containing approximately 91% protein, 7% lipid and 2% carbohydrates (mostly mannose in honeybee) in insects such as *A. mellifera* [41] and *Periplaneta americana* [42]; therefore, lipo- glyco- and phosphospecific staining of native gels were used to investigate purified Vg (Figure S2C–E). The bands at 200/180 and 46 kDa responded in the three different experiments indicating co-presence of those three posttranslational modifications on Vg-L and Vg-H. However, none of the stains showed a response on SDS-gels as observed for fish Vg earlier [43], a finding which raised doubt on the existence of covalent modifications. *Moxostoma hubbsi* Vg also exhibited poor staining after SDS-PAGE suggesting that the lipid content was reduced by the procedure [44]. On the other hand, it has not yet been clarified to what extend SDS masks the relevant moieties.

### Antibacterial activity of purified Vg

**Figure 1:**
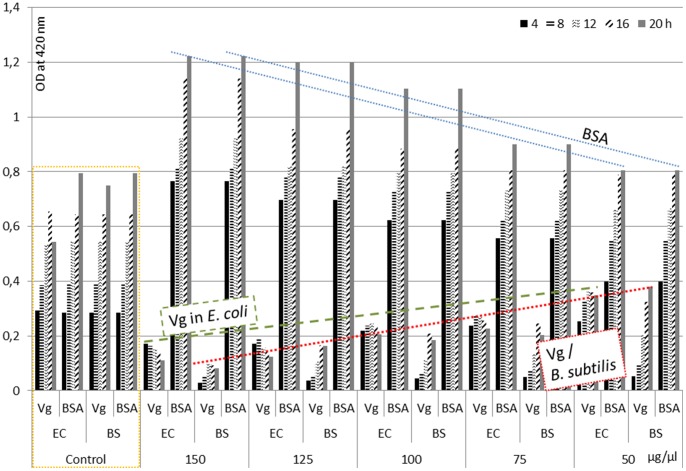
The antibacterial activity of purified Vg protein of *B. mori* against Gram negative (*E. coli*) bacteria and Gram positive (*B. subtilis*) bacteria. BSA was used as reference. Pure cell cultures were further controls.

The activity of purified Vg against Gram negative bacterium *E. coli* and Gram positive bacterium *B. subtilis* was analyzed by a time killing assay. *E. coli* and *B. subtilis* cultures were incubated with different concentrations of Vg protein for 24 h using BSA as control (Figure 1). E. *coli* growth was inhibited by all tested concentrations of Vg albeit to different extent. The lowest concentration (50 µg/ml) showed 20% of growth inhibition throughout the incubation period. 75 and 100 µg/ml of Vg concentration exhibited 30% of growth inhibition after 4 h incubation. Subsequently, the growth inhibition was increased by 60% and maintained through the rest of the incubation period. The highest concentration of Vg protein (125 and 150 µg/ml) showed 80% growth inhibition at the initial incubation period. However, when bacterial growth entered the lag phase, growth was inhibited up to 75%. The growth inhibition effect of Vg on *B. subtilis* was even higher. In both bacterial culture systems the addition of BSA supported cell growth; apparently it was a good nutrient source.

**Figure 2:**
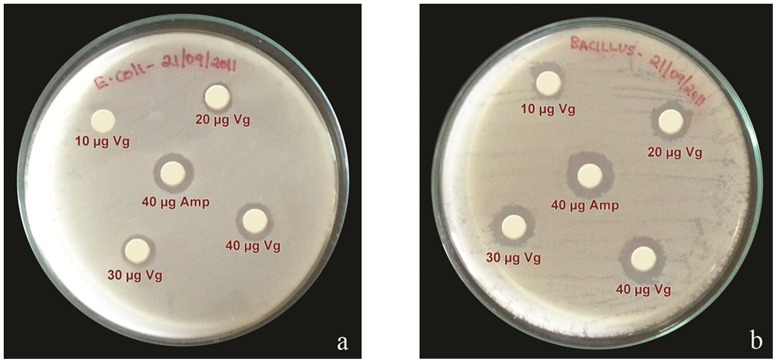
Sensitivity assay to examine the antibacterial activity of purified Vg protein against *E. coli* a) and *B. subtilis* b) bacteria. Ampicillin served as reference.

In order to test the anti-bacterial activity of Vg in comparison to the antibacterial agent ampicillin the paper disc diffusion method was used (Figure 2). In case of *E. coli*, 10 µg purified Vg did not inhibit bacterial growth, only higher amounts showed inhibition zones up to 12 mm (Table S1). In *B. subtilis* all applications of Vg exhibited a response as documented in inhibition zones of up to 14 mm.

**Figure 3:**
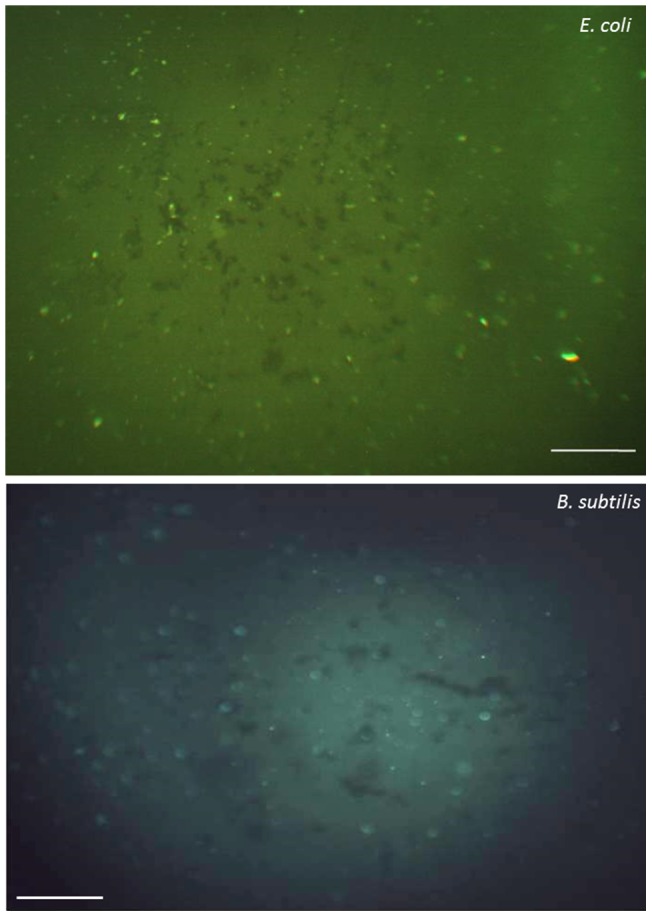
Binding of FITC-labeled Vg to *E. coli* (top) and *B. subtilis*. After washing with 25mM Tris buffer, the microbes were applied to microscope slides and observed under an Olympus fluorescence microscope. Images from the BSA control were completely black. Scale bars 40µm. For brightfield images see Figure S3.

FITC labeling was employed to explore the mechanism by which Vg inhibits bacterial growth (Figure 3, Figure S3). After washing and removal of unbound FITC-labeled proteins no signal was measured for the control BSA (black image, not shown), but fluorescence was detected in case of Vg and cells were highlighted indicating Vg interaction with bacterial cells. In particular, Vg fluoresced strongly when bound to the cell membrane of Gram negative bacteria. Such interaction was attributed earlier to the binding of positively charged Vg with negatively charged lipopolysaccharides on the outer bacterial surfaces [22]. Inferior interaction was observed for the binding of fish Vg with lipoteichoicacid from Gram positive bacteria, but reports showed its binding to β-1,3 glucan from eukaryotic fungi and laminaria from brown algae [21], [22], [45].

**Figure 4:**
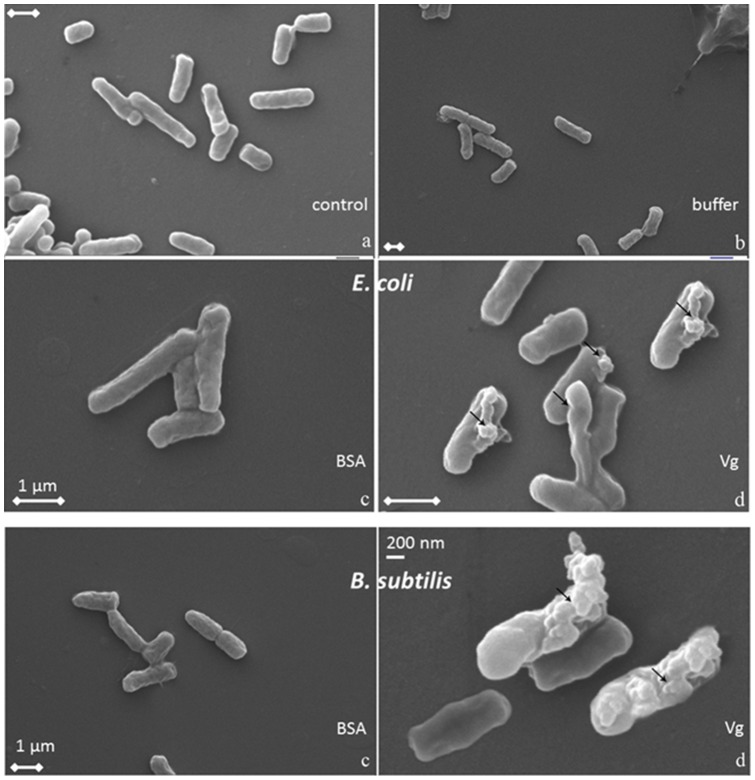
Scanning electron micrographs showing *E. coli* cells (top 4) and *B. subtilis* incubated with a) control, b) Tris citrate buffer, c) BSA, d) purified Vg. Arrows point to changes on the cell walls. The scale bars correspond to 1 µm unless stated otherwise.

SEM (Figure 4) visualized severe changes of the cell morphology for both types of bacteria indicating whole cell lysis. *B. subtilis* cells seem to respond more which is accordance with the antibacterial activity plate test described above. A similar effect had been published for phosvitin isolated from embryo of zebra fish showing the complete disintegration of both Gram negative and Gram positive bacteria [45].

**Figure 5:**
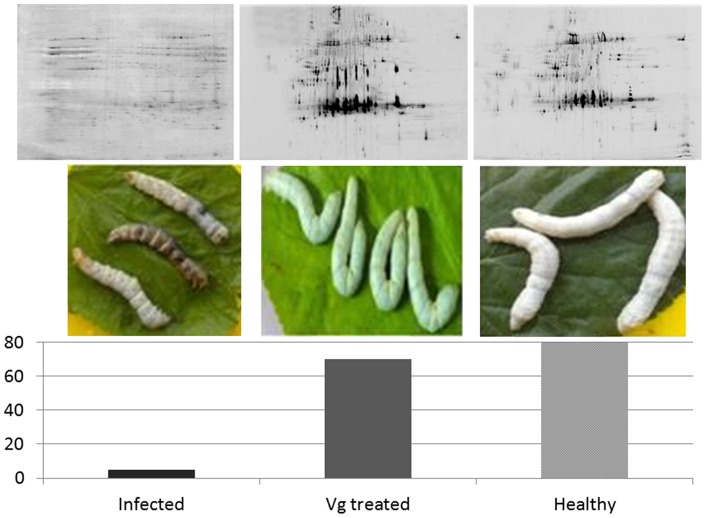
Therapeutic effect of Vg protein on silkworm (n = 75, Table S4) infected with *E. coli* and *B. subtilis* (in percent survival for two days). 200 µg of purified Vg was injected into infected silkworms for treatment. The healthy control group received a slit in the middle appendage with a sterile needle but no other treatment. Images were taken after 2 days. 2D-gel images (pH 3–10, MW 10–200kDa) of the respective hemolymph proteome are depicted on top.

Ultimately, purified Vg was injected into silkworm infected with *E. coli* and *B. subtilis* (Figure 5). While less than 10% of the untreated infected silkworm population lived for more than two days, 70% of the Vg-treated group survived a full life cycle similar to the healthy control group. This effect could be monitored not only by the appearance of the silkworm themselves but also in the hemolymph proteome pattern obtained by 2D-PAGE (Figure 5). The changes in the total proteome upon infection were quite striking. Several of the normally highly abundant proteins such as the 30 kDa lipoproteins [29] seemed drastically lowered in concentration, if not removed, from the proteome, possibly due to proteolytical degradation.

While antibacterial activity has already been attributed to Vg purified from barb [46], respective molecular mechanisms are still poorly understood as was reviewed by Jenssen *et al.* [47]. Liu *et al.* [22] reported that sequence analysis of Vg amino acids showed no similarity with “antibacterial peptide and lysozyme” but it remained unclear which antibacterial peptide was referred to. A database for antibacterial peptides is available administrating approximately 3000 sequences, not all of them based on experimental data [48], [49]. Nevertheless, we have performed a similarity analysis comparing VIT_BOMMO with the entire content of the database (Blast function of PLGS, Waters Corp.) and found matches for most of the Vg sequence. This result was in line with the realization that there are no general characteristic sequences for antimicrobial peptides apart from the observation that antimicrobial host defense peptides are known to adopt amphipathic conformations [47]. Therefore, it remains to be clarified which part of Vg could be responsible for the antimicrobial activity. It may not be the primary sequence, but rather protein decoration or both.

## Conclusion

For Vg purification, PVFB was collected from day 3 of pupa for best yield of intact Vg. Good separation of Vg from other molecules was achieved using anion-exchange chromatography, but three major protein forms were isolated as documented in gel bands at ∼46 and 180/200 kDa. Vg was identified in the bands as Vg-L and Vg-H, respectively, by MS and immunoblot. This conclusion was supported by native PAGE. The bands responded to specific tests for major posttranslational modifications indicating Vg’s nature as lipo-glyco-phosphoprotein. Dedicated analysis for specific protein modification sites needs to be performed in future experiments to describe the active structure of Vg.

Purified Vg exhibited antibacterial activity against Gram negative bacterium *E. coli* and Gram positive bacterium *B. subtilis*. The effect was entirely concentration dependent. Microscopic images proved binding of Vg to bacterial cells and their destruction. This observation suggested that Vg functioned as a pattern recognition molecule for the detection of bacteria. Pattern recognition receptors interacting with different saccharides moieties of microbes were already described in numerous invertebrate and vertebrate species [22]. When infected silkworm larvae were treated with Vg they survived the full life cycle in contrast to untreated animals (90% dead after 2 days). This result indicated that purified Vg has the ability to inhibit the proliferation of bacteria in the silkworm fluid system without disturbing the regular metabolism of the host. Similar results were observed when the infected silkworms were treated with antibiotics such as ampicillin, oxacillin or vancomycine [50]. The data presented above contribute to the understanding of the immunological role of yolk proteins in insect system.

## Supporting Information

Figure S1 **Electrophoretic profile and corresponding Western blot for Vg of PVFB collected from days 1 to 6 of V instar larvae.** (**V1–V6**, 7% SDS-PAGE, 50 µg/lane total protein) Immunoblot analysis was performed after protein transfer to a nitrocellulose membrane probing with primary antibody raised against Vg of *P. puparium*. Light purple staining indicated immunological cross reaction at ∼200 kDa from day 3 of V instar larvae. Staining was also observed at ∼200/180 and 46 kDa on day 6 of V instar larvae. Bands were tentatively assigned to Vg, Vg-H and Vg-L. **M –** MW marker.
(TIF)

Figure S2 **Purification and characterization of Vg protein.** **A**) Quality control using SDS-PAGE and silver staining after anion exchange chromatography (**L1-L6:** fraction 110–115. Three bands at ∼200, 180 and 46 kDa were tentatively labeled as Vg precursor, Vg-H, and Vg-L proteins, respectively. In fraction 115 the precursor was eluted resulting in two bands. **M –** MW marker. Native PAGE: Visible response was detected in the same regions with: **B**) Western blot of fraction 114 with primary antibody raised against Vg of *P. puparium*, **C**) Methyl green phosphoprotein staining of fraction 110, **D**) Periodic acid Schiff glycoprotein staining of fraction 110, **E**) Oil red ‘O’ lipoprotein staining of fraction 110.
(TIF)

Figure S3 **Binding of FITC-labeled Vg protein of** ***B. mori*** **to microbial cells – cells under bright field channels.** See Figure 3.
(TIF)

Table S1 **Zone of inhibition assay of Vg protein against** ***E. coli*** **and** ***B. subtilis*** **.** See Figure 2.
(DOC)

Table S2 **Survival of insects following treatment with** ***E. coli*** **and** ***B. subtilis*** **.** See Figure 5. Per experiment, 15 insects were used (75 per treatment).
(DOC)
