# Supplementary figures and images for: Vitellogenin from the Silkworm, Bombyx mori: An Effective Anti-Bacterial Agent
Source: PLoS One. 2013 Sep 13;8(9):e73005. doi: 10.1371/journal.pone.0073005 (PMC3772815; doi:10.1371/journal.pone.0073005)

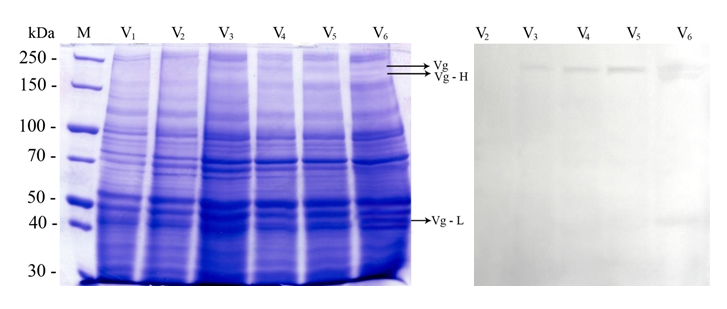

Supplement: Figure S1 — Electrophoretic profile and corresponding Western blot for Vg of PVFB collected from days 1 to 6 of V instar larvae. (V1–V6, 7% SDS-PAGE, 50 µg/lane total protein) Immunoblot analysis was performed after protein transfer to a nitrocellulose membrane probing with primary antibody raised against Vg of P. puparium. Light purple staining indicated immunological cross reaction at ∼200 kDa from day 3 of V instar larvae. Staining was also observed at ∼200/180 and 46 kDa on day 6 of V instar larvae. Bands were tentatively assigned to Vg, Vg-H and Vg-L. M – MW marker. (TIF) [file pone.0073005.s001.tif]

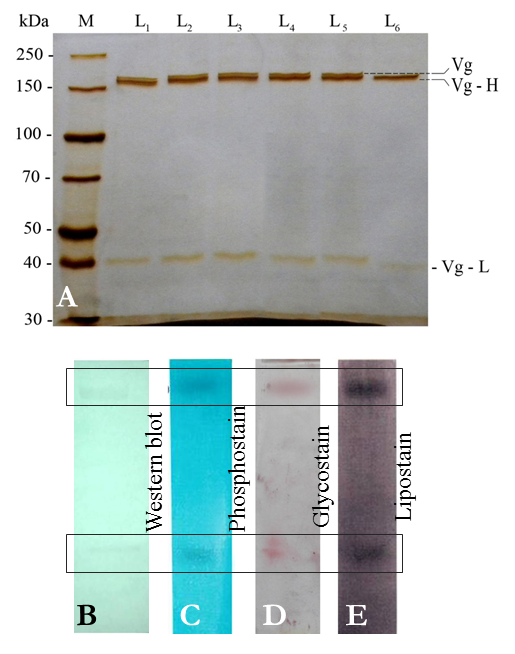

Supplement: Figure S2 — Purification and characterization of Vg protein. A) Quality control using SDS-PAGE and silver staining after anion exchange chromatography (L1-L6: fraction 110–115. Three bands at ∼200, 180 and 46 kDa were tentatively labeled as Vg precursor, Vg-H, and Vg-L proteins, respectively. In fraction 115 the precursor was eluted resulting in two bands. M – MW marker. Native PAGE: Visible response was detected in the same regions with: B) Western blot of fraction 114 with primary antibody raised against Vg of P. puparium, C) Methyl green phosphoprotein staining of fraction 110, D) Periodic acid Schiff glycoprotein staining of fraction 110, E) Oil red ‘O’ lipoprotein staining of fraction 110. (TIF) [file pone.0073005.s002.tif]

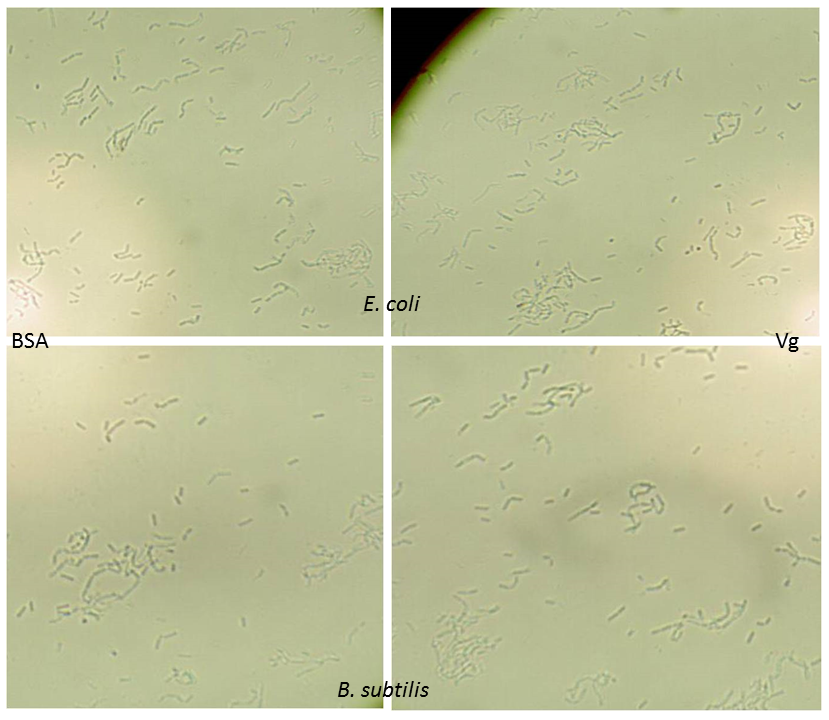

Supplement: Figure S3 — Binding of FITC-labeled Vg protein of B. mori to microbial cells – cells under bright field channels. See Figure 3. (TIF) [file pone.0073005.s003.tif]
