# Supplementary material for: Vitellogenin from the Silkworm, Bombyx mori: An Effective Anti-Bacterial Agent
Source: PLoS One. 2013 Sep 13;8(9):e73005. doi: 10.1371/journal.pone.0073005 (PMC3772815; doi:10.1371/journal.pone.0073005)
Supplement: Table S1 — Zone of inhibition assay of Vg protein against E. coli and B. subtilis . See Figure 2. (DOC) [file pone.0073005.s004.doc]

**Table S1:** Zone of inhibition assay of Vg protein against *E. coli* and *B. subtilis* (see Figure 2)

| **Organism** | **Zone of inhibition (mm)** | | | | |
| --- | --- | --- | --- | --- | --- |
|  | 40 µg Ampicillin | µg Vg | | | |
|  |  | 10 | 20 | 30 | 40 |
| *E. coli* | 12.2 | - | 3.2 | 8.6 | 11.5 |
| *B. subtilis* | 14.1 | 4.5 | 9.2 | 12.1 | 14.2 |
