# Supplementary material for: Vitellogenin from the Silkworm, Bombyx mori: An Effective Anti-Bacterial Agent
Source: PLoS One. 2013 Sep 13;8(9):e73005. doi: 10.1371/journal.pone.0073005 (PMC3772815; doi:10.1371/journal.pone.0073005)
Supplement: Table S2 — Survival of insects following treatment with E. coli and B. subtilis . See Figure 5. Per experiment, 15 insects were used (75 per treatment). (DOC) [file pone.0073005.s005.doc]

|  |  | | | | |
| --- | --- | --- | --- | --- | --- |
|  |  | | | |
|  |  |  |  |
|  |  |  |  |  |  |
|  |  |  |  |  |  |

**Table S2:** Survival of insects following treatment with *E. coli* and *B. subtilis* (see Figure 5). Per experiment, 15 insects were used (75 per treatment).

|  | **Living insects** | | |
| --- | --- | --- | --- |
| **Treatment** | **Bacteria** | **Bacteria+Vg** | **Control** |
| **Exp1** | 2 | 9 | 11 |
| **Exp2** | 3 | 11 | 12 |
| **Exp3** | 0 | 11 | 12 |
| **Exp4** | 0 | 10 | 13 |
| **Exp5** | 1 | 12 | 12 |
| **Total** | 6 | 53 | 60 |
| **%** | **8** | **70,7** | **80** |
| **Average** | 1,2 | 10,6 | 12 |
